# Treatment of displaced sagittal plane femoral condyle fractures (OTA 33 B1-2) with novel J-plate: technical trick and case series

**DOI:** 10.1007/s00590-026-04676-3

**Published:** 2026-02-19

**Authors:** Mitchel Obey, Brian Rust, David Barton, Jenna-Leigh Wilson, Marschall Berkes, Christopher McAndrew

**Affiliations:** Department of Orthopaedics, Washington University in St. Louis, St Louis, USA

**Keywords:** Distal femur fracture, Medial femoral condyle, Lateral femoral condyle, Unicondylar femur fracture

## Abstract

Surgical treatment of unicondylar distal femur fractures remains a challenge. Achieving stable fixation to allow early range of motion and weightbearing is complicated by poor bone quality and articular surface comminution, commonly leading to poor outcomes. Multiple treatment strategies have been previously described including small fragment, large fragment, and anatomical plate fixation. However, given the variability of fracture patterns, anatomical implants often lack the appropriate geometry and fixation options required to provide adequate stabilization. With knowledge and application of the principles of fracture fixation, these injuries can be effectively treated with standard non-anatomically shaped, contourable implants. The objective of this report was to describe a technique that provides effective fixation in these cases and report on outcomes of a small series. This technique utilizes a dual-implant construct that includes a small or large fragment plate positioned to buttress the metaphyseal apex of the fracture, and a small fragment reconstruction plate contoured in the shape of a “J” (J-plate) that wraps around the femoral condyle and onto the anterior cortex of the femur. There are advantages to this technique compared to anatomically contoured plates, including precise placement of implants in biomechanically advantageous locations, orthogonal fixation, and decreased implant costs.

## Introduction

Unicondylar fractures of the distal femur are rare injuries, accounting for less than 1% of all femoral fractures [1–4]. They typically occur following a direct impact onto a flexed knee, which includes low-energy falls in elderly patients and high-energy mechanisms in younger patients [5]. These fractures can occur in the lateral or medial femoral condyle, with fractures of the lateral condyle occurring more often. Fractures of the lateral condyle may be in the coronal plane (Hoffa fracture, OTA/AO 33B3) or the sagittal plane (OTA/AO 33B1) [6]. Whereas fractures of the medial condyle most commonly occur in the sagittal plane (OTA/AO 33B2), and very rarely in the coronal plane. Due to the intra-articular involvement and associated comminution of these fractures, increased rates of post-traumatic arthritis and malunion have been reported [1, 2, 5]. Furthermore, meniscal and cruciate ligament injuries have been reported in association with these injuries, which may further contribute to poor outcomes [1].

Nondisplaced patterns can be treated nonoperatively with good outcomes reported [1]. Most of these fractures, however, are indicated for surgical treatment especially if there is any significant degree of displacement or articular incongruity. Several internal fixation techniques have been previously described, yet there remains a paucity of literature regarding which is most optimal [1, 7–9]. Historically, the medial femoral condyle has been a location that lacked a reliable anatomically pre-contoured plate option. As a result, anatomic plates designed for other locations throughout the body such as the proximal tibia, proximal humerus, and distal tibia have been utilized for stabilizing these injuries [9, 10]. The availability of multiple anatomic plate options is highly dependent upon local hospital implant vendor contracts and implant costs, and surgeons therefore may be limited to standard small or large fragment implant sets when caring for these injuries.

The purpose of this article was to describe a surgical technique that provides rigid orthogonal fixation of unicondylar sagittal plane distal femur fractures and report on outcomes in a small case series. The technique utilizes a dual-implant construct with a small or large fragment plate, placed at the metaphyseal apex of the fracture, serving to buttress the fracture reduction. This is supplemented by a small fragment reconstruction plate contoured in the shape of a “J” (J-plate) that wraps around the femoral condyle and onto the anterior cortex of the femur. This allows for strategic and precise application of implants in biomechanically advantageous and orthogonal locations which increases construct stability. Supplemental fixation of the articular surface reduction can be implemented by the placement of independent lag screws or through the plates.

## Technique

**Fig. 1:**
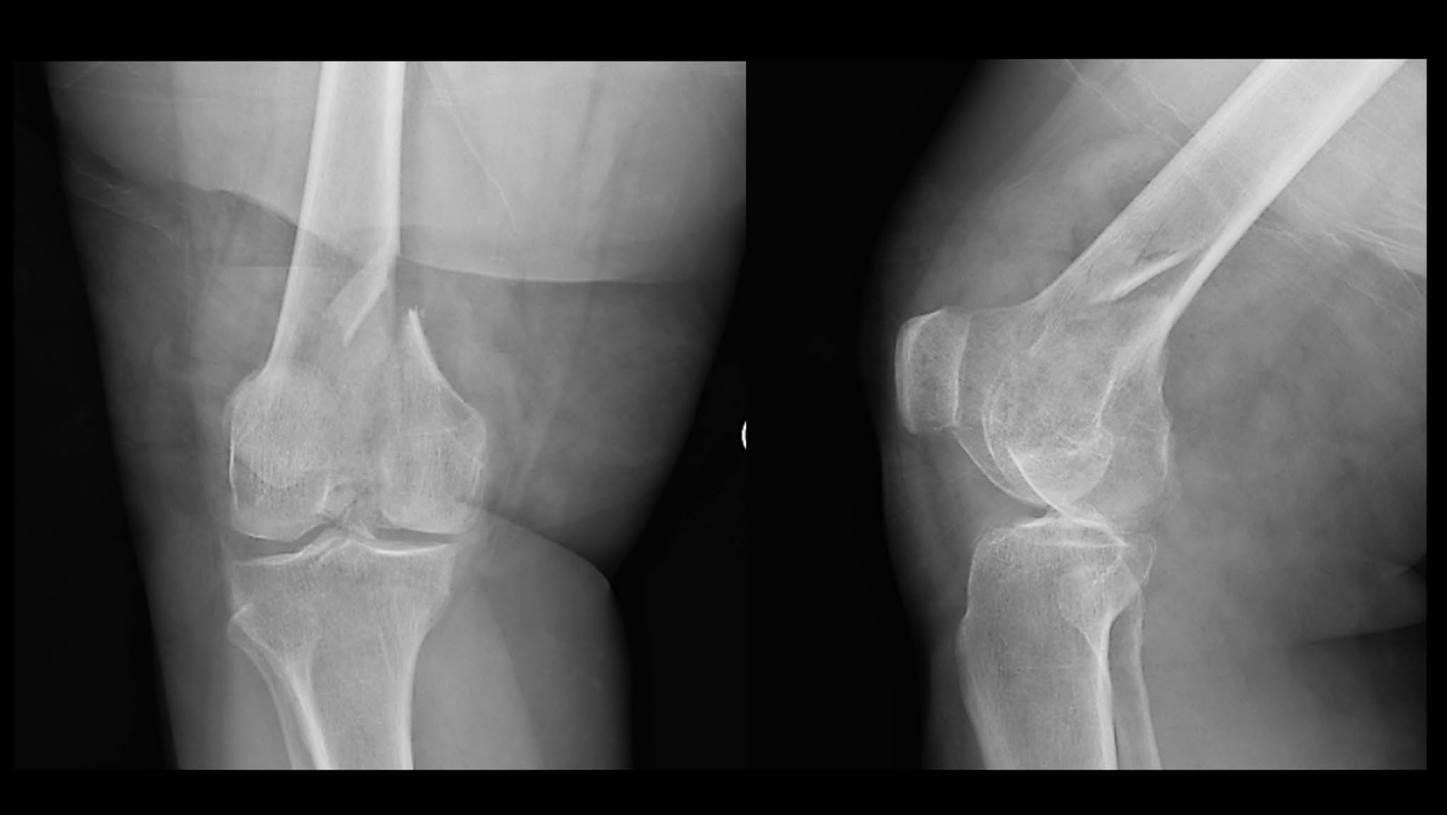
Injury anteroposterior and lateral radiogaphs of a comminuted medial femoral condyle fracture

**Fig. 2:**
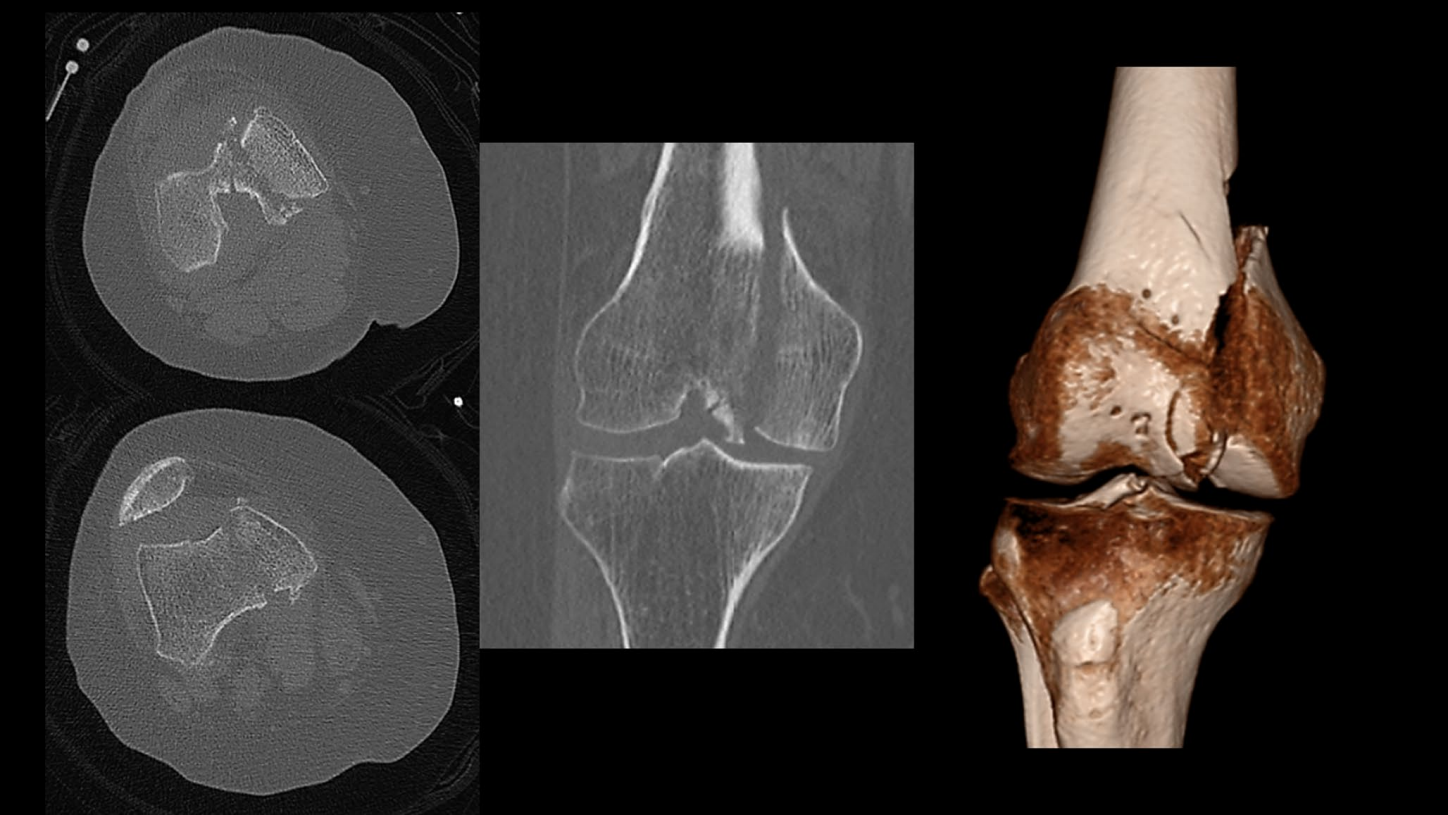
Injury computed tomography scan images and 3D reconstruction of a comminuted medial femoral condyle fracture

The surgical treatment utilizing a J-plate begins with patient positioning and exposure. Patients are positioned supine with the knee flexed over a radiolucent triangle. The exposure depends upon the fracture pattern. Fractures of the medial femoral condyle (Figs. 1 and 2) are approached through an anteromedial surgical incision that exposes the fracture through the vastus medialis and sartorius intermuscular interval. This incision can be extended distally to perform a medial parapatellar arthrotomy through which the joint can be debrided, and the articular fracture line can be visualized and reduced. Fractures of the lateral femoral condyle are approached through a direct lateral surgical incision that exposes the fracture through a lateral subvastus approach. Like the medial side, this incision can be extended distally to perform a lateral parapatellar arthrotomy for direct visualization and reduction of the articular fracture line.

Reduction of the metaphyseal fracture apex and articular surface can be facilitated with a distractor or external fixator, completed with direct clamp reduction, and stabilized with placement of smooth Steinmann pins or other techniques based on surgeon preference and fracture pattern. A series of fluoroscopic intraoperative images are then obtained to assess the reduction. These should include standard anteroposterior and lateral radiographs, as well as a femoral notch view which can be obtained by rotating the fluoroscopy machine caudally approximately 25 degrees.

**Fig. 3:**
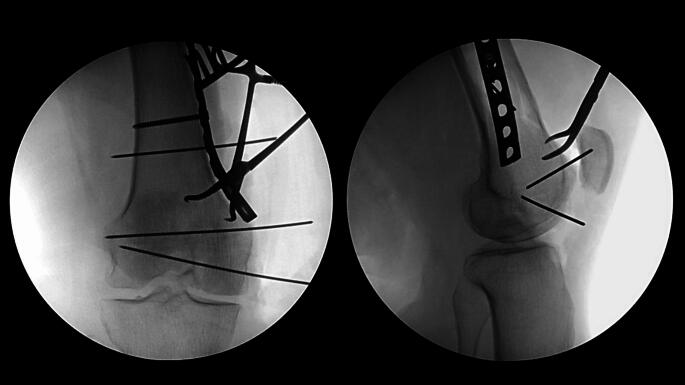
Intraoperative anteroposterior and lateral radiographs displaying buttress plate placement

**Fig. 4:**
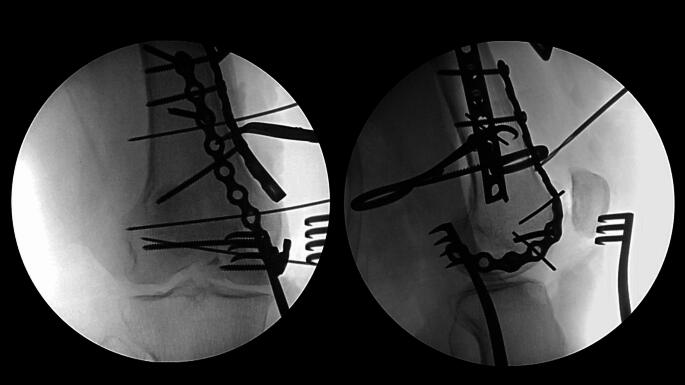
Intraoperative anteroposterior and lateral radiographs displaying J-plate placement

**Fig. 5:**
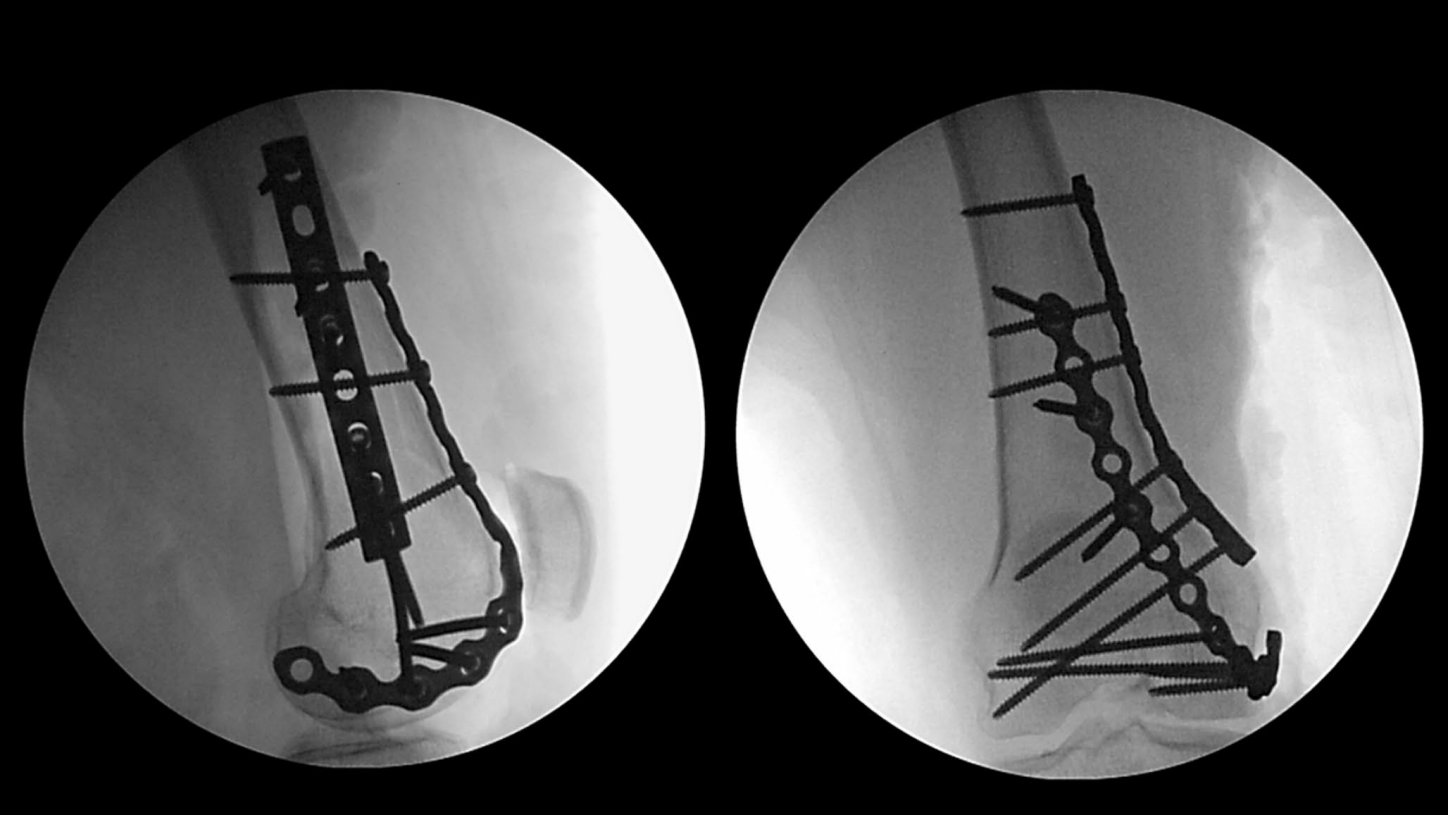
Final intraoperative anteroposterior and lateral radiographs displaying medial femoral condyle with near anatomic reduction

**Fig. 6:**
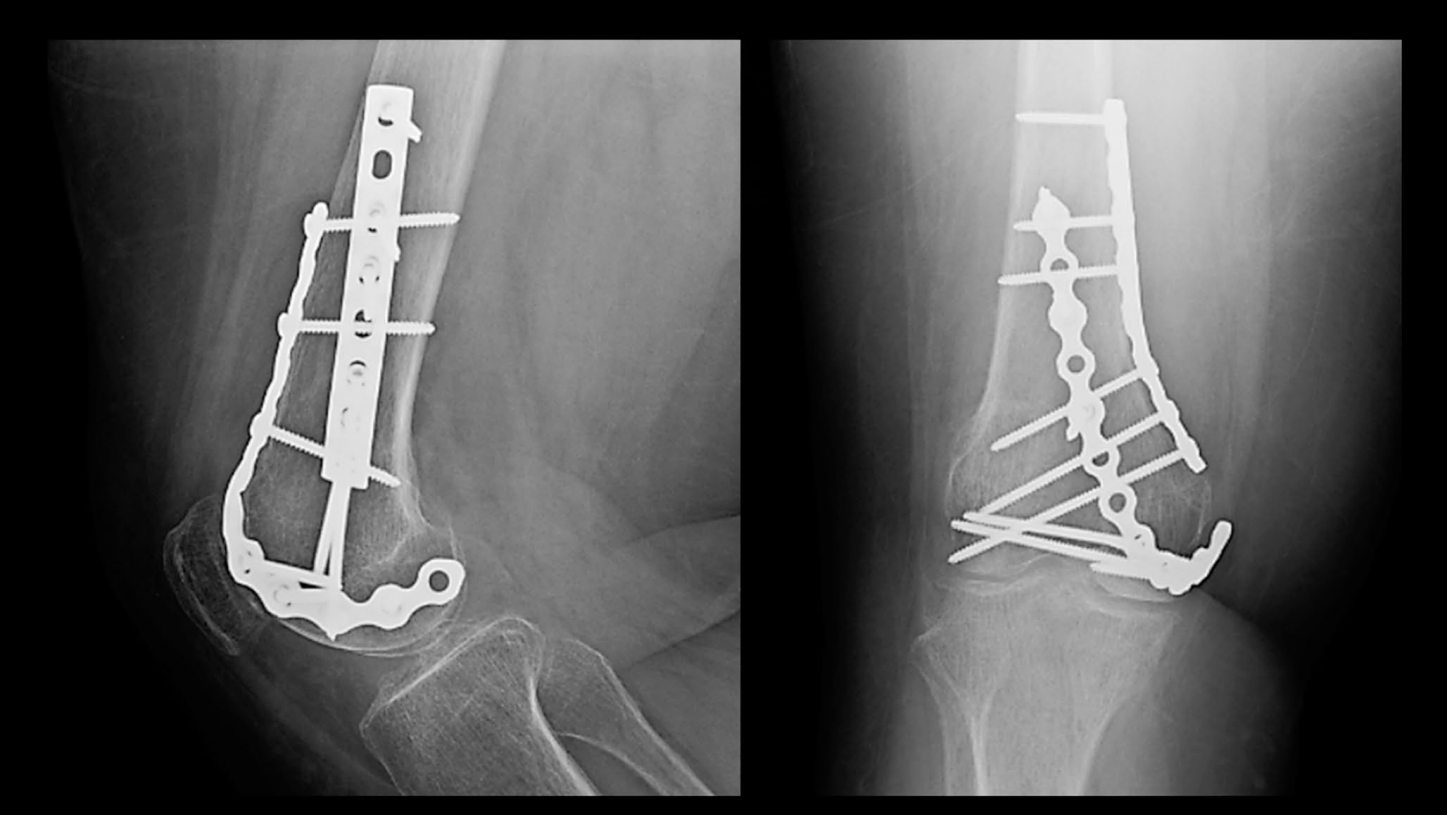
Final follow-up anteroposterior and lateral radiographs with healed fracture at 6 months postoperative

A small or large fragment LCP (locking compression plate) or DCP (dynamic compression plate) plate of the appropriate length as determined by the primary surgeon’s discretion is now selected, contoured, and centered over the metaphyseal fracture apex to serve as a buttress (Fig. 3). This plate is secured with the first available screw hole just proximal to the fracture apex. At this juncture, the surgeon can proceed with initial definitive fixation of the articular surface reduction with placement of independent lag screws if the bone quality and fracture pattern is amenable. A small fragment reconstruction plate of the appropriate length is now selected and contoured in the shape of a “J”, with the curve of the “J” wrapping around the periphery of the femoral condyle and twisting onto the anterior aspect of the distal femur (Fig. 4). The plate can be contoured with a variety of plate benders standardly found in small and large fragment implant trays. When doing this for the first time it is often helpful to utilize the plate bending templates found in the tray to guide contouring. The objective is to have the plate positioned along the periphery of the condyle distally which allows for placement of screws from medial to lateral (or vice versa) across the articular segment, using the plate as a cortical replacement in an anatomic area and typical patient scenario where the bone is intolerant of lag screw compression. As the plate extends proximally, it transitions to running along the anterior surface of the femur buttressing the anterior fracture extension and allowing for placement of screws in the orthogonal plane from anterior to posterior (Fig. 5). In addition, this plate can “connect” the anterior and posterior condyles in the setting of a coronal plane fracture. Postoperatively patients are kept non-weightbearing for 6 to 12 weeks depending on bone quality and allowed for immediate early range of motion in a hinged knee brace unlocked. Patients are then followed postoperatively for approximately 6 months or until fracture union (Fig. 6).

### Case series

A single center retrospective case series study was designed following institutional review board (IRB) approval using medical records and radiographs. Patients with a unicondylar sagittal plane distal femur fracture (OTA/AO B1-2) who were treated with a J-plate were identified and included in the study. Patients with isolated coronal plane Hoffa fractures (OTA/AO B3) were excluded from the study as well as those with no follow-up prior to confirmation of fracture union [11, 12]. Basic patient demographics, injury characteristics, and surgical data were collected, including: patient age and gender, body mass index, injury mechanism, fracture pattern including OTA/AO classification [6], type and size of plates used, and presence of independent lag screw fixation of the articular segment.

Postoperative follow-up data collected included weight bearing status, duration of follow-up in days, final follow-up knee range of motion, and fracture union. Complications were also collected, and these included infection or wound dehiscence, implant failure, loss of reduction, fracture malunion or nonunion, and any reoperations.

Twenty patients with 21 unicondylar sagittal plane distal femur fractures were treated with the J-plate technique by one of four total surgeons at a single institution from 2015 to 2025, and otherwise met inclusion criteria for the study. The mean patient age was 57 years (range, 27–79 years), mean BMI was 31.9, and patients were 67% female (14/21). Mean follow-up was 434.7 days (range, 24−3626 days) (Table 1). The average knee range of motion at time of final follow-up was 2.8 degrees short of full extension (range, 0–20) to 112.6 degrees of flexion (range, 70–135). The most common injury mechanism was a low-energy fall from standing (11/21), followed by motor vehicle crash (4/21), ballistic (4/21), bicycle crash (2/21). The majority of fractures involved the medial femoral condyle (13/21), and one patient sustained bilateral medial femoral condyle fractures. All fractures reached osseous union except for one patient who was lost to follow-up after their initial appointment 24 days following surgery.

**Table 1:** Patient and fracture demographics

	*n* = 21
Mean age, y.	57.0
*Sex*	
Male	7
Female	14
Mean BMI	31.9
*Femoral condyle*	
Medial	13
Lateral	8
*OTA/AO classification*	
33B1.1	3
33B1.2	1
33B1.3	4
33B2.1	7
33B2.2	1
33B2.3	5
*Injury mechanism*	
Standing level fall	11
Motor vehicle crash	4
Ballistic	4
Bicycle crash	2
*Buttress plate*	
Small fragment	73%
Large fragment	27%
J-plate	
Mini fragment	5%
Small fragment	95%

The surgical approaches used for fixation included an anteromedial approach for all medial femoral condyle fractures and a direct lateral subvastus approach for all lateral femoral condyle fractures. There were no patients that initially underwent closed reduction with knee-spanning external fixator application prior to definitive fixation. The plate types utilized for buttressing the fracture apex included no mini fragment plates, 73% with small fragment plates, and 27% with large fragment plates. Among the reconstruction plates that were contoured in the shape of a “J”, one patient was treated with a mini fragment 2.7 mm reconstruction plate, and the rest were 3.5 mm small fragment reconstruction plates. Independent lag screw fixation of the articular segment occurred in 23% of fractures (Table 1).

There were five complications observed. These included: one patient with a superficial wound infection that underwent surgical irrigation and debridement at 3 months postop; one patient who underwent an arthroscopic lateral meniscus debridement at 6 months postop; one patient who healed with a valgus malunion; one patient who healed with a varus malunion and subsequently underwent removal of implants and total knee arthroplasty at 5 years postop; and one patient who underwent removal of implants at 1 year postop for implant prominence.

## Discussion

In this article, the authors introduce a novel technique for internal fixation of displaced sagittal plane unicondylar fractures of the distal femur using standard non-anatomically contoured small and large fragment implants. The clinical results of 21 fractures that underwent open reduction and stabilization of a unicondylar distal femur fracture using the J-plate technique are described. We observed a high rate of union and maintenance of reduction, with a low rate of major complications.

Early studies reporting outcomes of these fractures treated with internal fixation described techniques with non-anatomically contoured plates applied in buttress fashion [1, 7, 8]. More recent studies have reported the use of anatomically contoured plates designed for the lateral proximal tibia, medial distal tibia, and proximal humerus [9]. Upadhyay et al. published the first study investigating the “best fit” for the medial distal femur in 18 different anatomical plates and found ipsilateral anterolateral proximal tibia plates with variable angle options provided the best fit for the medial femoral condyle [9]. Similar results were reported in a recent biomechanical study [10]. Anatomically pre-contoured implants designed for the medial distal femur exist, however, they are not available through all implant vendors. As such, surgeons may be limited by implant availability due to hospital implant contracts and must “improvise” with other available implant options. If utilizing anatomic plates designed for other regions of the body, this may lead to excessive plate contouring that may damage screw holes and compromise implant strength. This article introduces an internal fixation technique that can be successfully executed with simple small and large fragment non-anatomically contoured implant sets. This also provides the opportunity for precise placement of implants in biomechanically advantageous locations and orthogonal fixation. These are concepts familiar to most traumatologists and may improve efficiency of implant application.

There are limitations related to this study. As a retrospective case series, the results are directly limited by the amount of data available for review in the electronic medical record. Length of follow-up varied between patients, with at least one patient lost to follow-up after their first postoperative appointment. Patients are commonly followed until fracture union is achieved, which often occurred between 3 and 6 months. Without universal long-term follow-up, it is difficult to determine true rates of post-traumatic arthritis and the need for subsequent arthroplasty procedures. The longest follow-up in this study was observed in patients who later underwent total knee arthroplasties for complications, such as malunion or post-traumatic arthritis. A way to address this limitation for future study would be to ensure uniform long term follow-up with a prospective analysis. Also, future study would benefit from a larger cohort which could more accurately indicate the rates of complications and revisions. Another limitation of this study is related to the surgical technique, which likely differed between the four treating surgeons included in this study. It is difficult to discern the degree of impact this may have on the results, as all surgeons were fellowship trained traumatologists. Finally, nearly half (9/21) of the fractures included in this study were multifragmentary with involvement of the weight bearing articular surface (33B1.3 or 33B2.3). These injury patterns are more severe than simple fractures through the notch, and this may have negatively affected the outcomes.

In summary, the J-plate technique has demonstrated as an effective option for surgeons when treating displaced sagittal plane unicondylar fractures of the distal femur. The advantages of this technique include precise placement of implants in biomechanically advantageous locations, orthogonal fixation, and decreased implant costs.

## Data Availability

No datasets were generated or analysed during the current study.
